# The possible effect of inflammation on non-suicidal self-injury in adolescents with depression: a mediator of connectivity within corticostriatal reward circuitry

**DOI:** 10.1007/s00787-025-02709-6

**Published:** 2025-04-05

**Authors:** Dan Qiao, Yirun Qi, Xiaoyu Zhang, Yujiao Wen, Yangxi Huang, Yiran Li, Penghong Liu, Gaizhi Li, Zhifen Liu

**Affiliations:** Department of Psychiatry, First Hospital of Shanxi Medical University, No. 85 Jiefang South Road, Taiyuan, 030001 China

**Keywords:** Adolescent depression, Non-suicidal self-injury, Inflammation, Striatum, Reward circuit

## Abstract

Non-suicidal self-injury (NSSI) in adolescent depression is a prevalent and clinically significant behavior linked to dysregulated peripheral inflammation and corticostriatal circuitry dysfunction. However, the neuroimmune mechanisms bridging these systems remain poorly understood. Here, we combined peripheral cytokine profiling with static/dynamic functional connectivity (sFC/dFC) analysis to investigate the potential influence of inflammaton on corticostriatal circuit related to NSSI. A set of peripheral blood inflammatory markers and resting-state functional magnetic resonance imaging (rs-fMRI) were collected in depression with NSSI (NSSI+), depression without NSSI (NSSI-), and healthy controls (HC). We first ascertain group differences in level of pro- and anti-inflammatory cytokines. And using ventral/dorsal striatal seeds, we compared whole-brain, voxel-wise sFC and dFC differences across three groups. Further, we tested the mediation effects of connectivity in the association between inflammatory markers and NSSI frequency. NSSI+ group exhibited elevated pro-inflammatory cytokines (C-reactive protein (CRP), interleukin (IL)-1, and IL-6) whereas reduced anti-inflammatory cytokines (IL-10), compared to NSSI− and HC. Neuroimaging analysis revealed corticostriatal dysconnectivity mainly characterized by static hyperconnectivity between dorsal striatum and thalamus, dynamic instability in dorsal striatum-lingual pathways, and dynamic rigidity in ventral striatum-prefrontal/temporal/occipital gyrus circuits. Critically, sFC of dorsal striatum-thalamus and dFC of dorsal striatum-lingual gyrus mediated the prospective association between altered CRP and NSSI frequency, establishing corticostriatal circuits as conduits for inflammatory effects on NSSI. By bridging molecular psychiatry with circuit neuroscience, this work advances precision management of NSSI in adolescent depression, prioritizing biomarker-driven strategies to disrupt neuroimmune maladaptation.

**Supplementary Information:**

The online version contains supplementary material available at 10.1007/s00787-025-02709-6.

## Introduction

Depression in adolescence seriously endangers their mental and physical health, and is characterized by considerable heterogeneity in clinical manifestation [1, 2]. Converging lines of evidence suggest that non-suicidal self-injury (NSSI), defined as the intentional and self-inflicted damage to the body without suicidal intent, might account for a substantial disease burden in adolescents with depression [3]. Despite its clinical significance, the biological profile driving NSSI remain poorly understood, limiting the development of targeted interventions [4, 5].

Inflammation has emerged as one pathophysiologic pathway contributing to NSSI [6–8]. Adolescents with depression and NSSI exhibit dysregulated inflammatory profiles characterized by elevated pro-inflammatory markers (e.g., Interleukin (IL) −6 and C-reactive protein (CRP)) [9, 10] and deficient anti-inflammatory cytokines (e.g., IL-4, IL-13) [11]. Besides, longitudinal studies confirm that higher levels of IL-6 at baseline predict increased risk of NSSI at follow-up, further suggesting a causal role for inflammation in NSSI [10]. Critically, although there is a clear implication for inflammation in the pathoetiology of NSSI, the mechanism underlying this change and its involvement in NSSI among adolescents with depression remains elusive.

Emerging evidence positions that corticostriatal reward circuitry, a network governing reward learning, habit formation, and affect regulation, may serve as a potential conduit for the effect of inflammation on NSSI among adolescents with depression. This hypothesis is grounded in three interconnected lines of mechanistic and clinical evidence. First, pro-inflammatory cytokines directly suppress striatal dopamine synthesis by downregulating tyrosine hydroxylase activity, a rate-limiting enzyme in dopamine production, thereby disrupting reward prediction and reinforcement learning processes essential for adaptive decision-making [12–14]. Second, the adolescent developmental window is characterized by heightened neuroplasticity within corticostriatal circuits, which overlaps with a period of microglial priming and increased blood-brain barrier permeability, rendering these circuits uniquely sensitive to peripheral inflammatory signals [15, 16]. This confluence of developmental vulnerabilities may explain why inflammation during adolescence disproportionately increase susceptibility to maladaptive behaviors like NSSI compared to other life stages [17]. Finally, the operant conditioning mechanisms underlying NSSI, where self-injury is reinforced through immediate negative affect relief, align precisely with the corticostriatal circuit’s role in encoding reward-outcome associations [4, 18]. Neuroimaging and preclinical studies demonstrate that both opioid and dopamine signaling within the dorsal striatum mediate the transition from goal-directed to habitual behaviors, a pathway likely potentiated by inflammation-induced shifts in striatal neurochemistry [19–21]. Together, these mechanisms provide a neurobiologically coherent framework linking inflammation to NSSI via corticostriatal circuit dysfunction.

Moreover, neuroimaging studies to date has highlight the potential involvement of reward circuit in NSSI [4]. Specifically, Individuals with NSSI show blunted reward anticipation in fronto-limbic regions (e.g., amygdala, anterior cingulate cortex (ACC), and orbitofrontal cortex (OFC)) [22–24], yet paradoxically exhibit striatal hypersensitivity to immediate rewards (e.g., heightened putamen activation post-reward) [25]. This dissociation aligns with computational accounts of NSSI as a maladaptive habit: while fronto-limbic hypoactivity reflects impaired anticipation of rewards, striatal hyperactivity drives reinforcement of self-injury through negative affect relief [4]. Also, resting-state functional magnetic resonance imaging (rs-fMRI) literature further reveals aberrant amygdala connectivity with media prefrontal cortex (PFC)/ACC in NSSI [26, 27]. However, existing studies predominantly rely on static functional connectivity (sFC), which captures averaged connectivity strength over the entire scan, to characterize such circuit-level alterations. While sFC provides insights into stable, trait-like neural signatures, it overlooks the temporal dynamics of neural interactions that may be critical for understanding how inflammatory states modulate reward processing to trigger NSSI episodes. Dynamic functional connectivity (dFC), which quantifies moment-to-moment variability in neural coupling, offers a complementary lens to probe state-dependent neural effects [28]. For instance, acute inflammatory spikes could transiently disrupt brain communication, leading to abnormal performance that are missed by static analyses [29].

Altogether, despite growing interest in the role of inflammation in NSSI, little work has been done to link increased inflammation associated with changes in neurocircuitry that may precipitate risk of NSSI. Here, we integrate peripheral inflammatory profiling with neuroimaging data to test our hypotheses that NSSI-related inflammation correlates with aberrant functional connectivity (FC) within corticostriatal reward circuits among adolescents with depression. We first ascertain the aberrant level of peripheral pro-inflammatory and anti-inflammatory cytokines related to NSSI. And using voxel-wise analyses with ventral/dorsal striatum seeds, we map sFC and dFC patterns to NSSI and cytokine levels, while mediation models evaluate whether corticostriatal connectivity explains the inflammation-NSSI relationship. The findings may offer new perspectives for the management of NSSI, and further advance the development of personalized and precision treatment strategies for adolescents with depression.

## Methods

This cross-sectional study was approved by the Ethics Committee of the First Hospital of Shanxi Medical University with the approval number K-K040. All participants were informed of the nature, purpose, procedures, and potential risks and benefits of the study in detail. Especially, as part of the study procedures, participants will be required to provide about 5 ml of peripheral blood sample. This blood sample will be used for the inflammation analysis. And they were also informed that they would have the chance to receive a free magnetic resonance imaging (MRI) examination for precisely visualizing the brain regions. It should be emphasized that all their information will be strictly confidential and participants retain the right to withdraw from the study at any time. All participants and their guardians signed an informed consent form to participate in accordance with the ethical guidelines of the Declaration of Helsinki.

### Participants

From an initial cohort of 486 subjects aged 11–23 years (347 drug-naïve patients with first-episode depression and 139 healthy controls (HC)), the final analyzed sample sizes varied across study components due to data availability and quality control (QC) of MRI data. Part 1 analysis include 288 participants (185 patients and 103 HC), part 2 analysis include 342 participants (260 patients and 82 HC), and part 3 analysis include 165 participants (114 patients and 51 HC). The drug-naïve, first-episode depression participants were recruited from the Department of Psychiatry, First Hospital of Shanxi Medical University, Taiyuan, China. The HCs were recruited from Taiyuan, China, using advertisement in community. All participants were evaluated by two trained clinicians independently to determine the presence or absence of psychiatric diagnoses using the Mini International Diagnostic Interview (M.I.N.I).

Inclusion criteria for participants with depression were (1) major depressive disorder diagnosis according to the Diagnostic and Statistical Manual of Mental Disorders, Fifth Edition (DSM-5) criteria, (2) first episode, (3) no history of antidepressants, including pharmacological therapies (e.g., Selective Serotonin Reuptake Inhibitors, Serotonin-Norepinephrine Reuptake Inhibitors), psychotherapies (e.g., Cognitive Behavioral Therapy), and brain stimulation (e.g., transcranial magnetic stimulation and Electroconvulsive Therapy), (4) right-handedness, (5) ethnic Han. HCs had no history of any psychiatric diagnosis. Exclusion criteria for all participants were (1) the presence or history of any other major psychiatric disorders, severe medical, or neurological disorders, (2) experienced psychotic symptoms, (3) the presence of any acute and chronic infections, allergies, endocrine- or immune-related diseases, cancer, and systemic diseases, as determined by self-report or doctor’s report, (4) history of immunomodulatory treatment, analgesic/anti-inflammatory use, antibiotic therapy within 3 months, (5) contraindications to MRI scans, (6) Epileptic seizure history or family history of epileptic seizures.

### Clinical assessment

Participants completed a comprehensive diagnostic evaluation within 24 hours prior to the MRI scan. Demographic data were self-reported by the participants, including sex, age, and educational level. The severities of depressive symptom and anxiety symptom were assessed using the 24-item Hamilton Depression Rating Scale (HAMD-24) and 14-item Hamilton Anxiety Rating Scale (HAMA), respectively. NSSI was assessed with the Chinese version of the Ottawa Self-Injury Inventory (OSI). OSI is a self-rating scale comprising 28 items, designed to explore lifetime engagement in NSSI, the functions of NSSI, the level of addiction, and indicators of NSSI severity. Both the original and Chinese versions of this scale have demonstrated good test-retest reliability and construct validity in research and applications [30, 31]. We assessed whether adolescents engaged in NSSI behaviors according to the definition of NSSI in DSM-5 and related items in this scale. According to the presence or absence of NSSI, patients with depression were further divided into two groups: depression with NSSI (NSSI+) and depression without NSSI (NSSI-).

### Cytokines measures

Fasting peripheral venous blood samples (5 mL) were collected into EDTA test tubes by venipuncture in the morning. Consequently, the blood samples were centrifuged for 10 minutes at 3500 rpm, and the obtained plasma was kept frozen at −80°C until the analysis. Selected pro-inflammatory cytokines (IL-1, IL-2, IL-6, IL-12, IL-17, interferon-gamma (IFN-γ), tumor necrosis factor alpha (TNF-α), and CRP) and anti-inflammatory cytokines (IL-4 and IL-10) were analyzed and quantified in duplicate using Enzyme-Linked Immunosorbent Assays according to the procedures supplied by the manufacturer (Shanghai Jianglai Biotech, Shanghai, China). Before analysis, the values of all cytokines were log-transformed to improve normal distribution [32].

### Image acquisition

The structural and resting-state functional MRI data were collected at baseline, using a 3.0T MR system (Siemens Skyra) with a 20-channel birdcage head coil. Participants were required to keep their eyes closed but remain awake and relaxed throughout the entire scan, with restraining foam pads and rubber earplugs used to minimize head motion and noise interference. For each participant, a T1-weighted structural image was acquired using an MPRAGE Sagittal sequence (repetition time (TR) = 1900 ms, echo time (TE) = 3.97 ms, flip angle (FA) = 8°, acquisition matrix = 192 × 192, 192 slices, and slice thickness = 1 mm, with no gap). A whole-brain echo-planar imaging sequence was used to obtain functional data (TR/TE = 2620/30 ms, FA =90°, field of view = 192 × 192 mm, acquisition matrix = 64 × 64, slice thickness = 3 mm, with no gap, 47 slices, 220 volumes).

### Image data processing

The resting-state functional MRI data was analyzed with standard preprocessing protocols in Data Processing and Analysis for Brain Imaging software (DPABI; DPABI_V8.1_240101, http://rfmri.org/dpabi). Preprocessing steps mainly included: discard the first 10 volumes, slice-timing correction, realignment, co-registration to the corresponding T1 anatomical images, normalization, nuisance regression, spatial smoothing with a Gaussian kernel of 4 mm FWHM, linear detrending, and temporal band-pass filtering (0.01–0.08 Hz). Nuisance signals including the Friston-24 head motion parameters, white matter, and cerebrospinal fluid were regressed out for each individual. To minimize head motion effects, any subject with maximum head movement exceeding 2.0mm in displacement or more than 2.0° rotation was not included in the final analysis. The mean framewise displacement (FD) across time points of each subject were calculated. Furthermore, a “scrubbing” method at an FD threshold of 0.5 mm was utilized, with the “bad” time points and their 1-back and 2-forward neighbors estimated through linear interpolation.

Whole-brain resting-state connectivity was examined using 3-mm radius spherical seeds centered on ventral and dorsal striatal regions of interest (ROIs). We examined 4 subdivisions of striatum, including inferior ventral striatum (iVS), ventral rostral putamen (vrP), dorsal caudate (dC), and dorsal caudal putamen (dcP) (Table S1). These seeds are consistent with previously determined functional subregions of striatum [20, 33–35].

For sFC analysis, the mean blood oxygen level-dependent (BOLD) time course from each seed ROI was extracted and the Pearson’s correlation coefficients with the time course of all other voxels of the brain were calculated. Then, we transformed the correlation coefficients to Z values using the Fisher’s *r*-to-*z* transformation. To identify the dFC variability, a sliding window approach was applied with the Temporal Dynamic Analysis toolkit on DPABI. To avoid the introduction of spurious fluctuations, the minimum window length should be no less than 1/*f*_*min*_, which is deemed the minimum frequency of the time series [36, 37]. Based on this, a sliding-window length of 50 TRs and step size of 5 TRs were selected to calculate the temporal variability of connectivity. For each sliding window, the temporal correlation coefficient between the mean signals of the ROIs and all the other voxels in the whole-brain was computed. Then for each seed, the dFC maps were obtained for each participant. Consequently, all the dFC maps were converted into normal distribution by using the Fisher’s *r*-to-*z* transformation to the dFC values. For each individual, the dFC variance of the time series of each seed was measured by calculating the standard deviation of *z* value at each voxel to obtain the dFC variance map. Besides, considering the potential effect of window length on the dFC results, we further tested two window lengths (40 and 60 TRs) to validate the robustness of the dFC findings.

### Statistical analysis

We conducted the data analysis in three parts. First, we ascertain group differences in level of pro-inflammatory and anti-inflammatory cytokines among NSSI+, NSSI-, and HC groups in sample with blood data. Second, we examined sFC and dFC of each ORI differences among three groups in sample with neuroimaging data. Finally, we investigated the mediation effect of sFC/dFC in the association between inflammatory markers and NSSI in sample with both blood and neuroimaging data. Hence, samples in 3 parts are partially overlapping but not identical, which mitigates the risk of circularity. Specifically, for each step, demographic characteristics (age and educational years) and severity of clinical symptoms (HAMD-24 and HAMA-14 total score) were compared using a one-way analysis of variance (ANOVA). Categorical variables (sex) were compared using chi-squared test. A *P* value* <* 0.05 was considered as statistically significant.

To examine group differences in inflammatory markers and sFC/dFC while controlling for demographic and clinical confounders, we conducted analyses within the General Linear Model (GLM) framework.

In the first part of our investigation, to examine differences among the three groups, a one-way analysis of covariance (ANCOVA) was performed, adjusting for age, sex, and educational level. Post-hoc pairwise comparisons (NSSI+ *vs.* NSSI−, NSSI+ *vs.* HC, NSSI− *vs*. HC) were performed with Bonferroni-adjusted *P*-values to control Type I error. To assess whether demographic variables moderated group differences, interaction terms (Group × Age, Group × Sex, Group × Education) were added to the GLM. The significance of these interactions was evaluated using omnibus *F*-tests, with Bonferroni correction applied to three interaction terms (adjusted threshold: α = 0.05/3 = 0.017). Within the NSSI+ subgroup, partial correlation analyses explored associations between cytokine levels and NSSI frequency over one year, controlling for age, sex, educational level, and clinical symptom severity (HAMD-24 and HAMA-14 scores). A *P* value* <* 0.05 was considered as statistically significant.

In the second part, group differences in sFC and dFC were examined via ANCOVA, adjusting for age, sex, education, and mean FD. Gaussian Random Field (GRF) theory was applied for cluster-level multiple comparison correction (voxel-level threshold: *P* < 0.001; cluster-level threshold: *P* < 0.05). Brain clusters showing significant group differences were extracted as ROIs, and post-hoc pairwise comparisons (Bonferroni-adjusted) were performed to delineate contrasts among NSSI+, NSSI−, and HCs. Similarly, to test demographic moderation effects on functional connectivity, interaction terms (Group × Age, Group × Sex, Group × Education) were incorporated into the GLM, with Bonferroni correction setting the adjusted threshold to α = 0.017. Within the NSSI+ subgroup, partial correlations between ROI-based sFC/dFC and NSSI frequency were computed, controlling for age, sex, education, clinical symptom severity (HAMD-24 and HAMA-14 scores), and FD. A *P* value* <* 0.05 was considered as statistically significant.

In the last part, we conducted parallel mediation analysis with bootstrap resampling, which is a powerful method in finding the standard error of an estimate with no assumptions required of the sampling distribution [38]. Approaches to mediation indicate that a pairwise correlation among independent variable (*X*), proposed mediator (*M*), and dependent variable (*Y*) is a necessary pre-condition to proceed mediation analysis. Accordingly, to ensure NSSI-related cytokines and sFC/dFC identified in part 1 and part 2 analysis that met criteria for inclusion in subsequent mediation models, simple bivariate associations between variables (including NSSI frequency) were assessed using Pearson’s partial correlations, regressing out age, sex, education level, HAMD-24 scores, and HAMA-14 scores (for correlations involving sFC/dFC, and mean FD were also regressed out). Specifically, In PROCESS V4.1 for SPSS, mediation analyses were conducted using model 4 and bias-corrected 95 % confidence intervals (CIs) produced from 10,000 bootstrap resamples. The level of cytokines correlated to NSSI was entered as an independent variable, NSSI frequency of one year was entered as a dependent variable, and sFC/dFC linked to both NSSI and cytokines was entered simultaneously as the proposed mediator. Age, sex, educational level, HAMD-24 scores, HAMA-14 scores, and mean FD were included as covariates in mediation model.

## Results

### Sample characteristics

Table 1 shows the demographic and clinical characteristics of all subjects.

**Table 1:** Demographic and clinical characteristics of the sample

	Part 1: sample with blood data					Part 2: sample with imaging data					Part 3: sample with both blood and imaging data				
	NSSI+ (N=91)	NSSI- (N=94)	HC (n=103)	Statistics	P value	NSSI+ (N=143)	NSSI- (N=107)	HC (N=82)	Statistics	P value	NSSI+ (N=55)	NSSI- (N=59)	HC (N=51)	Statistics	P value
Demographic variables															
Age, mean (SD), years	15.84 (2.58)	17.15 (2.89)	18.86 (1.57)	F = 39.36	< 0.001	15.85 (2.44)	16.78 (2.87)	18.85 (1.86)	F = 38.75	< 0.001	15.67 (2.34)	16.98 (2.78)	18.71 (1.87)	F = 21.56	< 0.001
Sex, No. (%)															
Male	17 (18.68)	37 (39.36)	23 (22.33)	χ^2^ = 11.69	0.003	33 (23.08)	46 (39.32)	23 (28.05)	χ^2^ = 9.89	0.016	11 (20.00)	29 (49.15)	11 (21.57)	χ^2^ = 14.34	0.001
Female	74 (81.32)	57 (60.64)	80 (77.67)			110 (76.92)	71 (60.68)	59 (71.95)			44 (80.00)	30 (50.85)	40 (78.43)		
Educational level, mean (SD), years	10.04 (2.57)	11.22 (3.01)	13.25 (1.80)	F = 42.19	< 0.001	10.12 (2.51)	11.35 (2.83)	12.93 (1.99)	F = 32.79	< 0.001	9.84 (2.45)	11.54 (2.96)	12.69 (1.89)	F = 17.56	< 0.001
Clinical variables															
HAMD-24, score, mean (SD)	25.62 (5.90)	24.10 (6.53)	1.69 (2.50)	F = 656.03	< 0.001	26.98 (6.43)	24.22 (6.63)	1.86 (2.74)	F = 527.49	< 0.001	24.13 (6.56)	21.88 (6.11)	1.96 (3.84)	F = 242.97	< 0.001
HAMA, score, mean (SD)	19.11 (4.72)	16.95 (5.60)	1.08 (1.87)	F = 518.12	< 0.001	18.41 (5.57)	16.19 (4.88)	1.36 (2.34)	F = 364.93	< 0.001	16.44 (5.48)	14.61 (4.52)	1.42 (2.83)	F = 178.78	< 0.001

*NSSI* non-suicidal self-injury, *HAMD-24*, 24-item Hamilton depression rating scale, *HAMA* 14-item Hamilton anxiety rating scale

In part 1, inflammatory markers were available for N = 288 participants, comprising N = 91 NSSI+ cases (mean [SD] age, 15.84 [2.58] years, 17 [18.68] males, 74 [81.32] females), N = 94 NSSI- cases (mean [SD] age, 17.15 [2.89] years, 37 [39.36] males, 57 [60.64] females) and N = 103 HC (mean [SD] age, 18.86 [1.57] years, 23 [22.33] males, 80 [77.67] females). Significant age and educational level differences were observed among three groups, the NSSI+ patients showed the youngest and lowest educational level, followed by the NSSI- group and HC group. And the NSSI- group had a significantly higher proportion of males than the NSSI+ group and HC group. For clinical symptoms, the NSSI+ patients demonstrated the highest scores on the HAMD-24 and HAMA scores, followed by the NSSI− group and the HC group.

In part 2, after QC procedures, analysable MRI data were available for N = 342 participants, comprising N = 143 NSSI+ cases (mean [SD] age, 15.85 [2.44] years, 33 [23.08] males, 110 [76.92] females), N = 117 NSSI- cases (mean [SD] age, 16.78 [2.86] years, 46 [39.32] males, 71 [60.68] females) and N = 82 HC (mean [SD] age, 18.85 [1.86] years, 23 [28.05] males, 59 [71.95] females). Significant age and educational level differences were observed among three groups, the NSSI+ patients showed the youngest and lowest educational level, followed by the NSSI- group and HC group. And the NSSI- group had a significantly higher proportion of males than the NSSI+ group and HC group. For clinical symptoms, the NSSI+ patients demonstrated the highest scores on the HAMA scores, followed by the NSSI− group and the HC group. No differences in the HAMD-24 scores were observed between NSSI+ and NSSI- group, whereas both these two groups’ scores were higher than those in the HC group. There were no statistical differences among the three groups in terms of mean FD (*F* = 1.70, *P* = 0.184, mean [SD] FD: NSSI+ 0.06 [0.03], NSSI- 0.07 [0.03], HC 0.06 [0.02]).

In part 3, after QC procedures, analysable inflammatory markers and MRI data were available for N = 165 participants, comprising N = 55 NSSI+ cases (mean [SD] age, 15.67 [2.34] years, 11 [20.00] males, 44 [80.00] females), N = 59 NSSI- cases (mean [SD] age, 16.98 [2.78] years, 29 [49.15] males, 30 [50.85] females) and N = 51 HC (mean [SD] age, 18.71 [1.87] years, 11 [21.57] males, 40 [78.43] females). Significant age and educational level differences were observed among three groups, the NSSI+ patients showed the youngest and lowest educational level, followed by the NSSI- group and HC group. And the NSSI- group had a significantly higher proportion of males than the NSSI+ group and HC group. For clinical symptoms, no differences in the HAMD-24 and HAMA scores were observed between NSSI+ and NSSI- group, whereas both these two groups’ scores were higher than those in the HC group. And there were no statistical differences among the three groups in terms of mean FD (*F* = 0.49, *P* = 0.611, mean [SD] FD: NSSI+ 0.06 [0.03], NSSI- 0.07 [0.03], HC 0.06 [0.02]).

### Profile comparison and association with NSSI in peripheral cytokines (Part 1)

#### Case-control differences in peripheral cytokines

**Fig.1:**
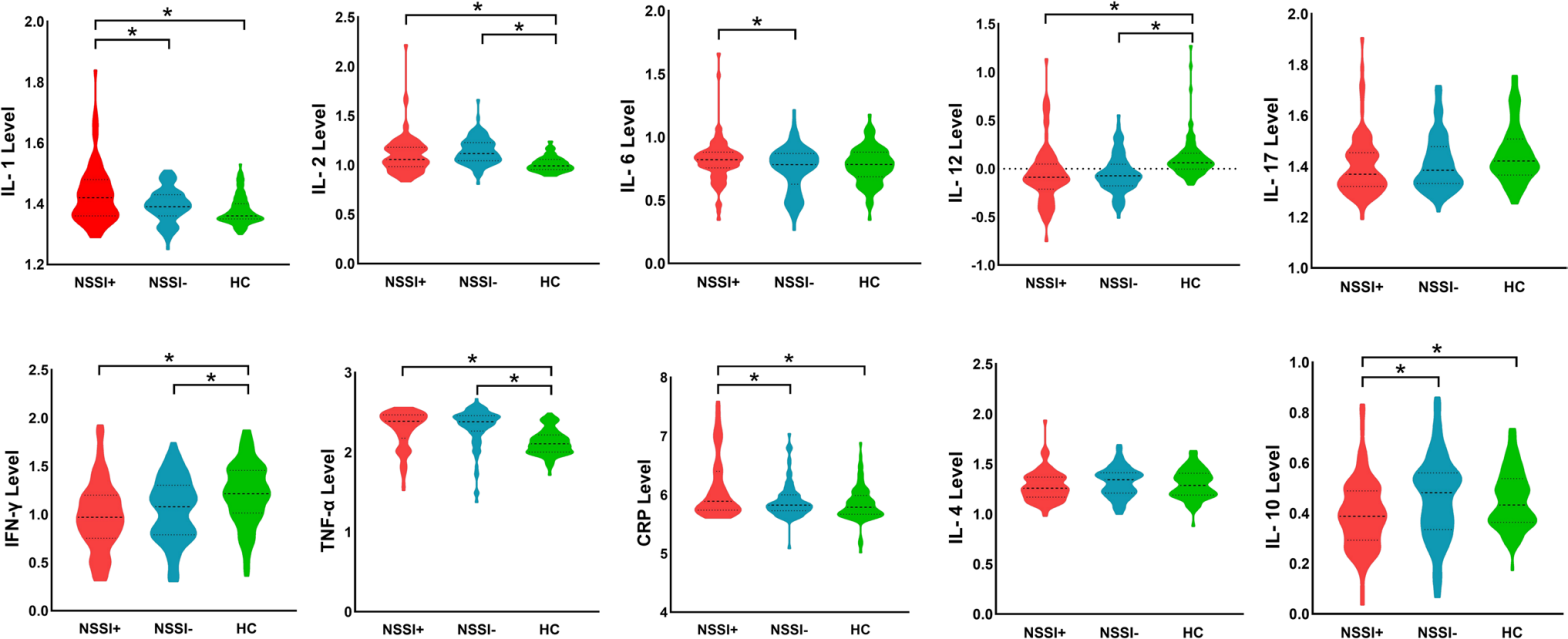
Profile comparison in peripheral cytokines

Age-, sex-, and educational level- adjusted ANCOVAs demonstrated that the three groups significantly differed in IL-1, IL-2, IL-6, IL-12, IL-17, IFN-γ, TNF-α, CRP, and IL-10 (all *P* < 0.05). In post hoc comparisons, the level of IL-6 in the NSSI+ group was higher than that in NSSI- group (*P* = 0.024, corrected by Bonferroni test), while no difference was observed between each patient group and HC group (all *P* > 0.05, corrected by Bonferroni test). Besides, the NSSI+ group had significantly higher concentration of IL-1 and CRP, as well as lower concentration of IL-10 (all *P* < 0.05, corrected by Bonferroni test), compared with both the NSSI- and HC groups, but no difference was observed between the NSSI- and the HC groups (all *P* > 0.05, corrected by Bonferroni test). Compared with the HC group, both the NSSI+ and NSSI- groups showed increased level of IL-2, IL-12, IFN-γ, and TNF-α (all *P* < 0.05, corrected by Bonferroni test), while no difference was observed between patient groups (all *P* > 0.05, corrected by Bonferroni test) (Fig. 1, Table 2). The omnibus *F*-tests revealed no significant interaction effects between demographic variables (age, sex, education) and groups (NSSI+, NSSI−, HC) for any cytokine (all *P* > 0.05, Bonferroni-adjusted α = 0.017) (Table S2).

**Table 2:** Case-control differences in inflammatory markers

	NSSI+ (N = 91)	NSSI- (N = 94)	HC (N = 103)	Statistics	*P* value	*P* value of Post-hoc analysis		
						NSSI+ *vs* NSSI-	NSSI+ *vs* HC	NSSI- *vs* HC
Pro-inflammatory and cytokines								
IL-1	1.43 (0.10)	1.39 (0.06)	1.38 (0.05)	12.38	< 0.001	0.003	< 0.001	0.385
IL-2	1.10 (0.19)	1.14 (0.13)	1.01 (0.08)	22.18	< 0.001	0.182	< 0.001	< 0.001
IL-6	0.82 (0.17)	0.75 (0.18)	0.77 (0.15)	3.60	0.029	0.024	0.314	0.805
IL-12	−0.13 (0.42)	−0.13 (0.34)	0.11 (0.21)	17.173	< 0.001	0.901	< 0.001	< 0.001
IL-17	1.43 (0.17)	1.41 (0.11)	1.45 (0.13)	1.87	0.155	0.674	1.000	0.170
IFN-γ	0.98 (0.35)	1.05 (0.33)	1.21 (0.32)	12.37	< 0.001	0.586	< 0.001	0.002
TNF-α	2.27 (0.34)	2.32 (0.24)	2.12 (0.16)	16.64	< 0.001	0.500	< 0.001	< 0.001
CRP	6.11 (0.53)	5.90 (0.30)	5.84 (0.29)	12.89	< 0.001	< 0.001	< 0.001	0.864
Anti-inflammatory cytokines								
IL-4	1.28 (0.15)	1.32 (0.15)	1.30 (0.14)	1.81	0.166	0.179	0.763	1.000
IL-10	0.40 (0.14)	0.46 (0.17)	0.45 (0.12)	5.57	0.004	0.009	0.016	0.787

All results are shown as mean (SD) and expressed as pg/mL

*NSSI* Non-suicidal self-injury, *IL* Interleukin, *IFN-γ* Interferon-gamma, *TNF-α* Tumor necrosis factor alpha, *CRP* C-reactive protein

#### Association between peripheral cytokines and NSSI in patients with NSSI

Correlation analysis in the NSSI+ group revealed positive correlations between NSSI frequency of one year and CRP level (*r* = 0.738, *P* < 0.001).

### Profile comparison and association with NSSI in sFC and dFC (Part 2)

#### Case-control differences in sFC

**Fig.2:**
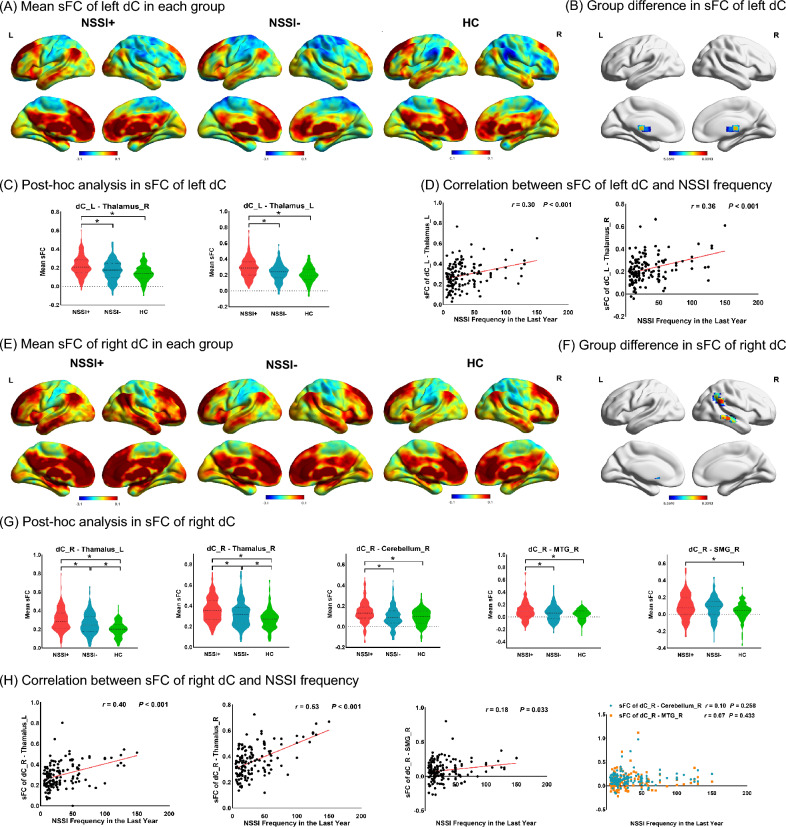
Profile comparison and association with NSSI in sFC

For sFC, age-, sex-, educational level-, and mean FD- adjusted ANCOVAs demonstrated that the three groups significantly differed in sFC between the left dC and the bilateral thalamus (left: *F* = 9.30, *P* < 0.001; right: *F* = 9.21, *P* < 0.001) (Fig. 2A and B, Table S3), as well as altered sFC between the right dC and the bilateral thalamus (left: *F* = 12.45, *P* < 0.001; right: *F* = 13.49, *P* < 0.001) , the right supramarginal gyrus (SMG) (*F* = 8.75, *P* < 0.001), the right cerebellum (*F* = 11.52, *P* < 0.001), and the right middle temporal gyrus (MTG) (*F* = 8.56, *P* < 0.001) (Fig. 2E and F, Table S3). In post hoc comparisons, the NSSI+ group had significantly increased sFC between the left dC and the bilateral thalamus (left: NSSI+ *vs* NSSI-: *P* < 0.001, NSSI+ *vs* HC: *P* < 0.001, NSSI- *vs* HC: *P* = 0.222; right: NSSI+ vs NSSI-: *P* = 0.001, NSSI+ *vs* HC: *P* < 0.001, NSSI- *vs* HC: *P* = 0.112, corrected by Bonferroni test), as well as increased sFC between the right dC and the right MTG (NSSI+ *vs* NSSI-: *P* = 0.030, NSSI+ *vs* HC: *P* < 0.001, NSSI- *vs* HC: *P* = 0.120, corrected by Bonferroni test) and the right cerebellum (NSSI+ *vs* NSSI-: *P* = 0.001, NSSI+ *vs* HC: *P* = 0.002, NSSI- *vs* HC: *P* = 1.000, corrected by Bonferroni test) compared with both the NSSI- and HC groups, but no difference was observed in sFC between the NSSI- and the HC groups. Besides, group comparison of sFC between the right dC and the bilateral thalamus revealed hierarchical decrease pattern, with NSSI+ showing highest connection, followed by NSSI- cases, and then HC (left: NSSI+ *vs* NSSI-: *P* = 0.023, NSSI+ *vs* HC: *P* < 0.001, NSSI- *vs* HC: *P* = 0.007; right: NSSI+ *vs* NSSI-: *P* = 0.018, NSSI+ *vs* HC: *P* < 0.001, NSSI- *vs* HC:* P* = 0.006, corrected by Bonferroni test) (Fig. 2C and G). The omnibus *F*-tests revealed no significant interaction effects between demographic variables (age, sex, education) and groups (NSSI+, NSSI−, HC) for any sFC (all *P* > 0.05, Bonferroni-adjusted α = 0.017) (Table S2).

#### Association between sFC and NSSI in patients with NSSI

Correlation analysis in the NSSI+ group revealed positive correlations between NSSI frequency of one year and sFC of left dC - bilateral thalamus (left: *r* = 0.35, *P* < 0.001; right: *r* = 0.30, *P* < 0.001), right dC - bilateral thalamus (left: *r* = 0.40, *P* < 0.001; right: r = 0.53, *P* < 0.001), and right dC - right SMG (*r* = 0.18, *P* = 0.033) (Fig. 2D and H).

#### Case-control differences in dFC

**Fig.3:**
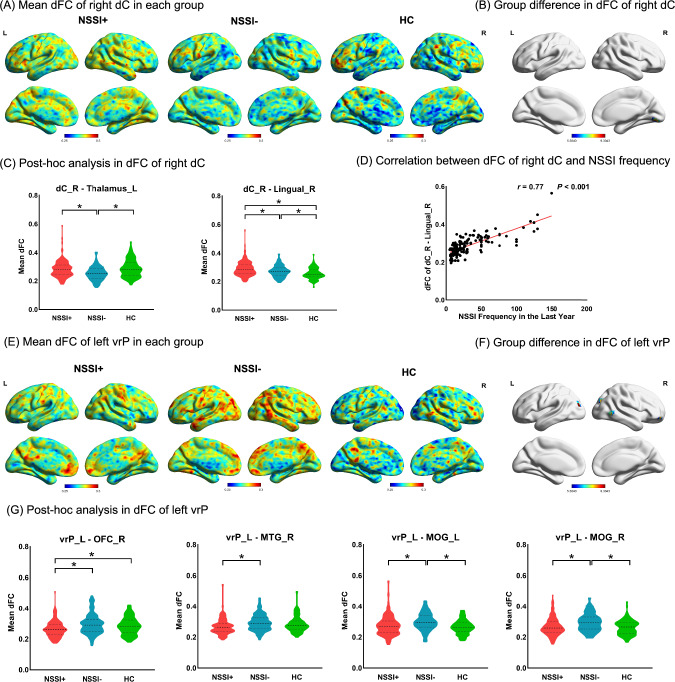
Profile comparison and association with NSSI in dFC

For dFC, age-, sex-, educational level-, and mean FD- adjusted ANCOVAs demonstrated that the three groups significantly differed in dFC between the right dC and the right lingual gyrus (*F* = 10.05, *P* < 0.001), and the left thalamus (*F* = 10.21, *P* < 0.001) (Fig. 3A and B, Table S3), as well as altered dFC between the left vrP and the bilateral middle occipital gyrus (MOG) (left: *F* = 10.46, *P* < 0.001; right: *F* = 9.37, *P* < 0.001), right MTG (*F* = 8.47, P < 0.001), and right OFC (*F* = 8.24, *P* < 0.001) (Fig. 3E and F, Table S3). In post hoc comparisons, NSSI+ group showed higher dFC between the right dC and the right lingual gyrus (NSSI+ *vs* NSSI-: *P* = 0.008, NSSI+ *vs* HC: *P* < 0.001, NSSI- *vs* HC: *P* = 0.038, corrected by Bonferroni test), as well as lower dFC between the left vrP and the right OFC (NSSI+ *vs* NSSI-: *P* < 0.001, NSSI+ *vs* HC: *P* = 0.046, NSSI- *vs* HC: *P* = 0.548, corrected by Bonferroni test), compared to the NSSI- group and HC group. Besides, NSSI+ group showed higher dFC between the right dC and the left thalamus (NSSI+ *vs* NSSI-: *P* < 0.001, NSSI+ *vs* HC: *P* = 0.451, NSSI- *vs* HC: *P* < 0.001, corrected by Bonferroni test), as well as decreased dFC between the left vrP and the bilateral MOG (left: NSSI+ *vs* NSSI-: *P* < 0.001, NSSI+ *vs* HC: *P* = 0.362, NSSI- *vs* HC: *P* < 0.001; right: NSSI+ *vs* NSSI-: *P* < 0.001, NSSI+ *vs* HC: *P* = 0.574, NSSI- *vs* HC: *P* < 0.001, corrected by Bonferroni test), and right MTG (NSSI+ *vs* NSSI-: *P* < 0.001, NSSI+ *vs* HC: *P* = 0.056, NSSI- *vs* HC: *P* = 0.427, corrected by Bonferroni test) (Fig. 3C and G). The omnibus *F*-tests revealed no significant interaction effects between demographic variables (age, sex, education) and groups (NSSI+, NSSI−, HC) for any dFC (all *P* > 0.05, Bonferroni-adjusted α = 0.017) (Table S2).

#### Association between dFC and NSSI in patients with NSSI

Correlation analysis in the NSSI+ group revealed positive correlations between NSSI frequency of one year and dFC of right dC - right lingual gyrus (*r* = 0.767, *P* < 0.001) (Fig. 3D).

#### Validation analysis in dFC

Validation analyses using alternative sliding-window lengths (40 and 60 TRs) revealed that the group difference in dFC of right dC - left thalamus was not statistically significant, while all other dFC findings consistent with the primary analysis (Table S4, Figure S1−2).

### Mediation analysis (Part 3)

**Fig.4:**
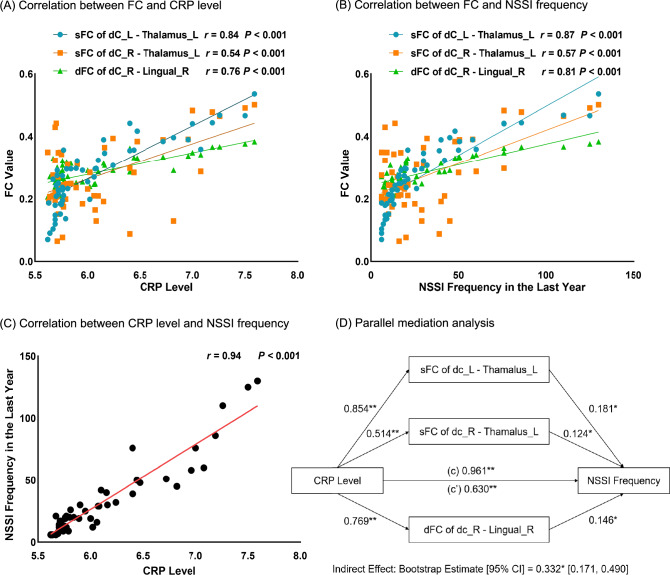
Correlation analysis and mediation analysis in NSSI+ group

Figure 4A-C shows the correlation analysis of associated factors among NSSI+ group. The concentration of CRP, sFC/dFC (i.e., sFC of bilateral dC - left thalamus and dFC of right dC - right lingual gyrus), and NSSI frequency were mutually correlated with each other. Parallel mediation analysis showed that sFC of bilateral dC - left thalamus and dFC of right dC - right lingual gyrus were significant mediators for the relation of CRP level and NSSI frequency (Fig. 4D). The sFC of bilateral dC - left thalamus and dFC of right dC - right lingual gyrus mediated 34.49% of the total effect of CRP level on NSSI frequency. Of these, the mediating effects of sFC between left dC - left thalamus, sFC between right dC - left thalamus, and dFC between right dC - right lingual gyrus account for 16.13%, 6.67%, and 11.69% of the total effect of CRP level on NSSI frequency, respectively.

## Discussion

This study provides the first integrative evidence linking peripheral inflammation, corticostriatal circuit dysfunction, and NSSI in adolescents with depression. The main significant results can be summarized as follows: (1) Elevated pro-inflammatory cytokines (CRP, IL-1, IL-6) and reduced anti-inflammatory IL-10 distinguish adolescents with NSSI from those without NSSI and HC, (2) NSSI is associated with hyperconnectivity in dorsal striatum-thalamus/lingual pathways and hypoconnectivity in ventral striatum-OFC/MTG/MOG circuits. (3) aberrant connectivity within corticostriatal circuitry could mediate the relationship between inflammation and NSSI frequency, suggesting a "brain-immune" pathway underlying self-injurious behaviors.

### Aberrant level of inflammatory marker in adolescent depression with NSSI

Pro-inflammatory cytokines are signaling proteins in the body that typically trigger inflammatory reactions and tend to stimulate immunocompetent cells, whereas anti-inflammatory cytokines inhibit inflammation and suppress immune cells [39], both can also alter the metabolism of key monoamines that are involved in the pathogenesis of depression in aolescence [6, 40]. The findings from the current study add to the literature supporting a potential risk pathway between inflammation and NSSI in depression. Specifically, we found NSSI is characterized by enhanced pro-inflammatory CRP, IL-1, and IL-6 signaling associated impaired IL-10-linked anti-inflammatory activity. The dysregulated pro-/anti-inflammatory balance in NSSI aligns with prior reports linking inflammation to maladaptive coping behaviors [10]. Elevated CRP and IL-6 correlate with symptoms of anhedonia and stress-induced pain [20, 41, 42], which may drive individuals toward NSSI as a maladaptive coping mechanism [43]. Conversely, reduced IL-10, a cytokine critical for suppressing excessive inflammation, may exacerbate neural sensitivity to negative feedback, further predisposing adolescents to self-injury [44, 45]. These findings advocate for integrating routine inflammatory profiling (e.g., CRP screening) into psychiatric assessments to identify high-risk subgroups, which could be prioritized for anti-inflammatory interventions.

### Altered connectivity within corticostriatal circuitry in adolescent depression with NSSI

Based on functional evidence, the striatum can be divided into dorsal and ventral components with distinct roles. The dorsal striatal neuroadaptations have been implicated in the transition between incentive-based and habit-based control of behavior [46, 47]. Static hyperconnectivity between the dorsal striatum and thalamus, temporal gyrus, and cerebellum aligns with prior work [48, 49] implicating this circuit in compulsive and addictive behaviors. In general, aversion to pain dissuades most individuals from engaging in NSSI, and the erosion of this instinctive barrier in patients with depression may facilitate NSSI [50]. Given the role of the thalamus in pain-processing [51], and the effects of temporal gyrus and cerebellar activity on emotion-regulating [52, 53], increased connectivity with the dorsal striatum is likely associated with enhanced reward experience of painful self-administered stimulus, may be reinforcing NSSI as a maladaptive coping strategy. Notably, dynamic hyperconnectivity in the dorsal striatum-lingual pathway suggests heightened neural flexibility during reward processing. The lingual gyrus, involved in visual-spatial integration [54], may link self-injury imagery (e.g., viewing wounds) to dorsal striatal reward signals, perpetuating NSSI through visual-cue conditioning [48, 55]. This dynamic instability could reflect a failure to habituate to aversive stimuli, a hallmark of addiction-like behaviors. Our findings extend addiction models to NSSI, proposing that dorsal striatal hyperconnectivity, both static (trait-like) and dynamic (state-dependent), facilitates the transition from voluntary to habitual self-injury.

In contrast, the ventral striatum, a key node in reward valuation and motivational salience, showed reduced dFC variability with the OFC, MTG, and MOG in NSSI. Dynamic rigidity, defined as restricted moment-to-moment fluctuations in neural coupling, suggests impaired flexibility in integrating reward signals with contextual, emotional, and perceptual inputs. Specifically, diminished dFC variability of ventral striatum-OFC aligns with theories of NSSI as a failure to adaptively weigh long-term consequences against immediate relief [23]. The OFC, which updates value representations based on changing contingencies, may become "uncoupled" from ventral striatal reward signals during stress, locking individuals into maladaptive decision-making [56, 57]. Besides, the MTG and MOG, involved in social cognition and visuospatial processing [52, 58, 59], normally contextualize rewards within social or environmental frameworks. Reduced dFC variability in these pathways may disconnect NSSI from its social repercussions, perpetuating NSSI as a solitary coping strategy.

While dorsal striatal hyperconnectivity reflects habit-driven compulsivity, ventral striatal rigidity underscores reward-context uncoupling in NSSI. This dissociation parallels addiction literature, where dorsal striatum mediates habitual drug-seeking, whereas ventral striatum- prefrontal cortex dysconnectivity underpins poor impulse control [60, 61]. In NSSI, dynamic rigidity may similarly decouple reward valuation from inhibitory signals, enabling self-injury to persist despite negative outcomes.

### Mediation effect of functional connectivity in the association between inflammatory markers and NSSI

Critically, our mediation analyses bridge peripheral inflammation with brain dysfunction, proposing a neuroimmune model where corticostriatal circuits serve as conduits for inflammatory effects on behavior. Specifically, the correlation between CRP and NSSI frequency is driven by the strength of dorsal striatal and thalamus connectivity, as well as the variability of dorsal striatal and lingual gyrus connectivity, suggesting that systemic inflammation amplifies habit-forming neural loops. Although it is not clear weather CRP itself can traverse or signal across the blood brain barrier (BBB) [62], this model dovetails with clinical evidence showing that the corticostriatal reward circuit serves as a bridge for increased concentration of CRP and abnormal motivation, which was related to NSSI [20, 63, 64]. Hence, while the exact mechanisms of peripheral immune-to-brain signaling remain unclear, our findings underscore the dorsal striatum’s centrality in translating inflammatory states into behavioral pathology. Clinically, dual-target therapies simultaneously addressing inflammation and neural circuits may yield synergistic benefits.

### Strengths and limitations

The current study advances our understanding of NSSI in adolescent depression by establishing a novel neuroimmune model that integrates peripheral inflammation with corticostriatal circuit dysfunction. Two key innovations distinguish this work: first, by synergizing cytokine profiling with sFC/dFC analyses, we provide the first direct evidence that elevated pro-inflammatory markers correlate with both trait-like and state-dependent neural signatures in NSSI. Next, the study demonstrates that corticostriatal dysconnectivity mediates the relationship between inflammation and NSSI frequency, suggesting neural circuits through which peripheral immune signals translate into maladaptive behavior.

However, the study must be interpreted in the context of its limitations. First, although we found preliminary evidence that aberrant connectivity within corticostriatal circuitry could mediate indirect effects of inflammation on NSSI, causality cannot be inferred from our current cross-sectional study, further longitudinal studies will clearly be needed to validate this mechanistic model. Additionally, the modest sample size and demographic differences between patients and HC may limit generalizability. However, we mitigate confounding by rigorously controlling for these variables, and the convergence of inflammatory, neuroimaging, and behavioral data strengthens mechanistic plausibility. Future work should prioritize large-scale cohorts with serial assessments to validate our neuroimmune model and explore potential modifiers of inflammation-brain interactions.

## Conclusions

This study pioneers a neuroimmune model of NSSI in adolescent depression by integrating multimodal biomarkers, peripheral inflammation and connectivity within corticostriatal circuit, to unravel how systemic immune signals translate into self-injurious behaviors. Building on this framework, we propose a mechanism-guided intervention strategy that synergistically combines anti-inflammatory therapies with connectivity-targeted neuromodulation. This dual-target approach aims to disrupt neuroimmune maladaptation at both molecular and circuit levels, offering a transformative pathway to mitigate NSSI by concurrently resolving inflammation and corticostriatal circuitry dysfunction.

## Supplementary Information

Below is the link to the electronic supplementary material.

Supplementary file1 (DOCX 13358 KB)

## Data Availability

The data that support the findings of this study are not openly available due to reasons of sensitivity and are available from the corresponding author upon reasonable request.
