# Supplementary material for: The possible effect of inflammation on non-suicidal self-injury in adolescents with depression: a mediator of connectivity within corticostriatal reward circuitry
Source: Eur Child Adolesc Psychiatry. 2025 Apr 5;34(9):2871–85. doi: 10.1007/s00787-025-02709-6 (PMC12508009; doi:10.1007/s00787-025-02709-6)
Supplement: Supplementary file 1 — Supplementary file1 (DOCX 13358 KB) [file 787_2025_2709_MOESM1_ESM.docx]

**Table S1**. Coordinates for ventral and dorsal striatal regions of interest.

| ROIs | MNI Space Coordinates |
| --- | --- |
| ventral striatum |  |
| inferior ventral striatum (iVS) | ±14, 8, -9 |
| ventral rostral putamen (vrP) | ± 20, 12, -3 |
| dorsal striatum |  |
| dorsal caudal putamen (dcP) | ± 28, 1, 3 |
| dorsal caudate (dC) | ±13, 15, 9 |

**Table S2**. Group × Demographic Interactions (Bonferroni-Adjusted α = 0.017)

|  | Group × Age | | Group × Sex | | Group × Education | |
| --- | --- | --- | --- | --- | --- | --- |
|  | *F* value | *P* value | *F* value | *P* value | *F* value | *P* value |
| pro-inflammatory and cytokines | | | | | | |
| IL-1 | 1.76 | .174 | 0.78 | .458 | 2.27 | .105 |
| IL-2 | 1.67 | .189 | 0.55 | .525 | 0.65 | .575 |
| IL-6 | 0.05 | .954 | 0.27 | .761 | 0.03 | .972 |
| IL-12 | 1.15 | .317 | 1.57 | .211 | 0.40 | .669 |
| IL-17 | 1.94 | .146 | 0.51 | .601 | 1.29 | .278 |
| IFN-γ | 2.70 | .069 | 1.77 | .172 | 2.73 | .067 |
| TNF-α | 1.37 | .326 | 0.02 | .984 | 0.38 | .700 |
| CRP | 0.50 | .607 | 0.88 | .417 | 0.27 | .757 |
| anti-inflammatory cytokines | | | | | | |
| IL-4 | 0.71 | .491 | 0.38 | .686 | 1.98 | .141 |
| IL-10 | 0.67 | .515 | 0.72 | .488 | 1.40 | .249 |
| sFC | | | | | | |
| dc_L - Thalamus_R | 0.87 | .422 | 1.10 | 333 | 0.19 | .829 |
| dc_L - Thalamus_L | 0.01 | .996 | 0.88 | .414 | 0.08 | .922 |
| dc_R - Thalamus_R | 0.65 | .523 | 0.24 | .785 | 0.92 | .399 |
| dc_R - Thalamus_L | 0.61 | .546 | 0.25 | .777 | 1.64 | .291 |
| dc_R - MTG_R | 1.16 | .315 | 0.30 | .738 | 0.29 | .746 |
| dc_R - Cerebelum_R | 0.34 | .711 | 1.20 | .301 | 0.11 | .892 |
| dc_R - SMG_R | 0.48 | .624 | 1.03 | .360 | 0.43 | .654 |
| dFC | | | | | | |
| dc_R - Lingual_R | 0.33 | .722 | 1.42 | .243 | 0.52 | .593 |
| dc_R - Thalamus_L | 1.09 | .337 | 0.69 | .501 | 0.29 | .751 |
| vrP_L - OFC_R | 0.82 | .442 | 0.29 | .746 | 0.61 | .545 |
| vrP_L - MTG_R | 0.27 | .761 | 1.28 | .278 | 0.43 | .649 |
| vrP_L - MOG_L | 0.44 | .646 | 0.41 | .667 | 0.86 | .425 |
| vrP_L - MOG_R | 0.04 | .966 | 0.30 | .742 | 0.09 | .911 |

sFC, static functional connectivity; dFC, dynamic functional connectivity; dC, dorsal caudate; vrP, ventral rostral putamen; MTG, middle temporal gyrus; SMG, supramarginal gyrus; OFC, orbitofrontal cortex; MOG, middle occipital gyrus

**Table S3**. Brain regions showing significant sFC and dFC differences across the three groups.

| ROIs | Region | Voxels | MNI Space Coordinates | | | *F* value |
| --- | --- | --- | --- | --- | --- | --- |
|  |  |  | X | Y | Z |  |
| sFC | | | | | | |
| dc_L | Thalamus_R | 54 | 12 | 0 | 6 | 9.21 |
|  | Thalamus_L | 94 | -12 | 0 | 6 | 9.30 |
| dc_R | Thalamus_R | 98 | 12 | 9 | 6 | 13.49 |
|  | Thalamus_L | 139 | -12 | 3 | 6 | 12.45 |
|  | MTG_R | 64 | 60 | -30 | -9 | 8.56 |
|  | Cerebelum_R | 443 | 3 | -90 | -30 | 11.51 |
|  | SMG_R | 71 | 57 | -45 | 27 | 8.75 |
| dFC | | | | | | |
| dc_R | Lingual_R | 32 | 18 | -75 | -9 | 10.05 |
|  | Thalamus_L | 10 | -6 | -15 | 9 | 10.21 |
| vrP_L | OFC_R | 13 | 36 | 57 | -12 | 8.24 |
|  | MTG_R | 16 | 60 | -60 | 0 | 8.47 |
|  | MOG_L | 19 | -27 | -81 | 21 | 10.45 |
|  | MOG_R | 20 | 27 | -75 | 30 | 9.37 |

sFC, static functional connectivity; dFC, dynamic functional connectivity; dC, dorsal caudate; vrP, ventral rostral putamen; MTG, middle temporal gyrus; SMG, supramarginal gyrus; OFC, orbitofrontal cortex; MOG, middle occipital gyrus.

**Table S4**. Brain regions showing significant dFC differences across the three groups in validation analysis.

| ROIs | Region | Voxels | MNI Space Coordinates | | | *F* value |
| --- | --- | --- | --- | --- | --- | --- |
|  |  |  | X | Y | Z |  |
| Window length: 40TRs | | | | | | |
| dc_R | Lingual_R | 23 | 18 | -75 | -9 | 9.14 |
| dc_R | OFC_R | 13 | 36 | 57 | -12 | 7.98 |
|  | MTG_R | 11 | 60 | -57 | 3 | 7.11 |
|  | MOG_L | 18 | -27 | -81 | 21 | 9.64 |
|  | MOG_R | 34 | 27 | -75 | 30 | 9.46 |
| Window length: 60TRs | | | | | | |
| dc_R | Lingual_R | 29 | 18 | -75 | -9 | 9.94 |
| vrP_L | OFC_R | 10 | 36 | 57 | -12 | 7.76 |
|  | MTG_R | 17 | 60 | -60 | 0 | 8.69 |
|  | MOG_L | 15 | -27 | -81 | 21 | 10.50 |
|  | MOG_R | 16 | 27 | -75 | 30 | 8.91 |

dFC, dynamic functional connectivity; dC, dorsal caudate; vrP, ventral rostral putamen; MTG, middle temporal gyrus; SMG, supramarginal gyrus; OFC, orbitofrontal cortex; MOG, middle occipital gyrus.


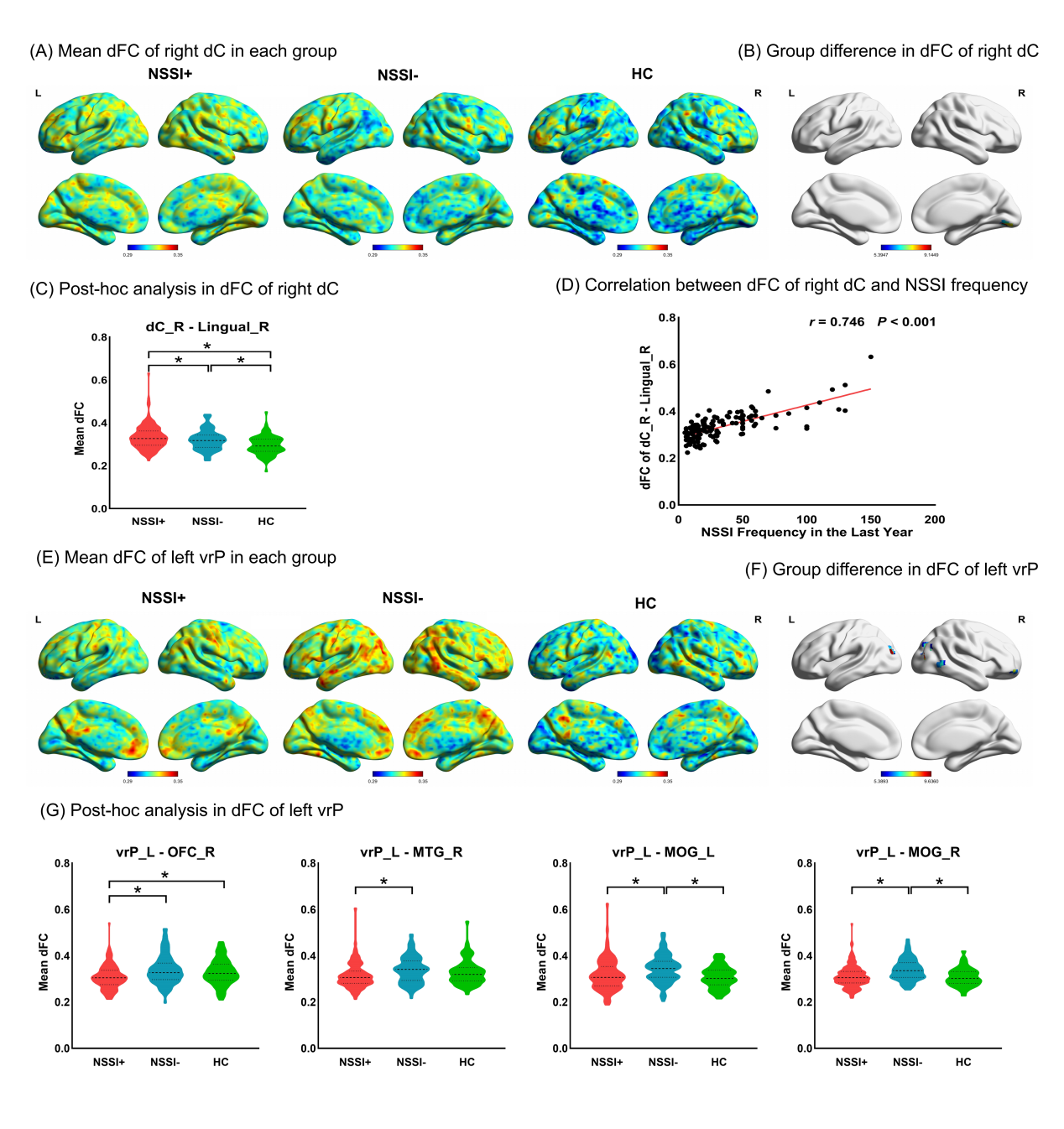


Figure S1. Profile Comparison and Association with NSSI in dFC in validation analysis (Window length: 40TRs). (A) The mean dFC map of right dC in each group. (B) Regions showing group difference in dFC of right dC. (C) Post-hoc analysis of regions with within-group differences in dFC of right dC. (D) Correlation between dFC of right dC and NSSI frequency. (E) The mean dFC map of left vrP in each group. (F) Regions showing group difference in dFC of left vrP. (G) Post-hoc analysis of regions with within-group differences in dFC of left vrP. *Bonferroni corrected, *P* < 0.05. dFC, dynamic functional connectivity; NSSI, non-suicidal self-injury; dC, dorsal caudate; vrP, ventral rostral putamen; OFC, orbitofrontal cortex; MTG, middle temporal gyrus; MOG, middle occipital gyrus.


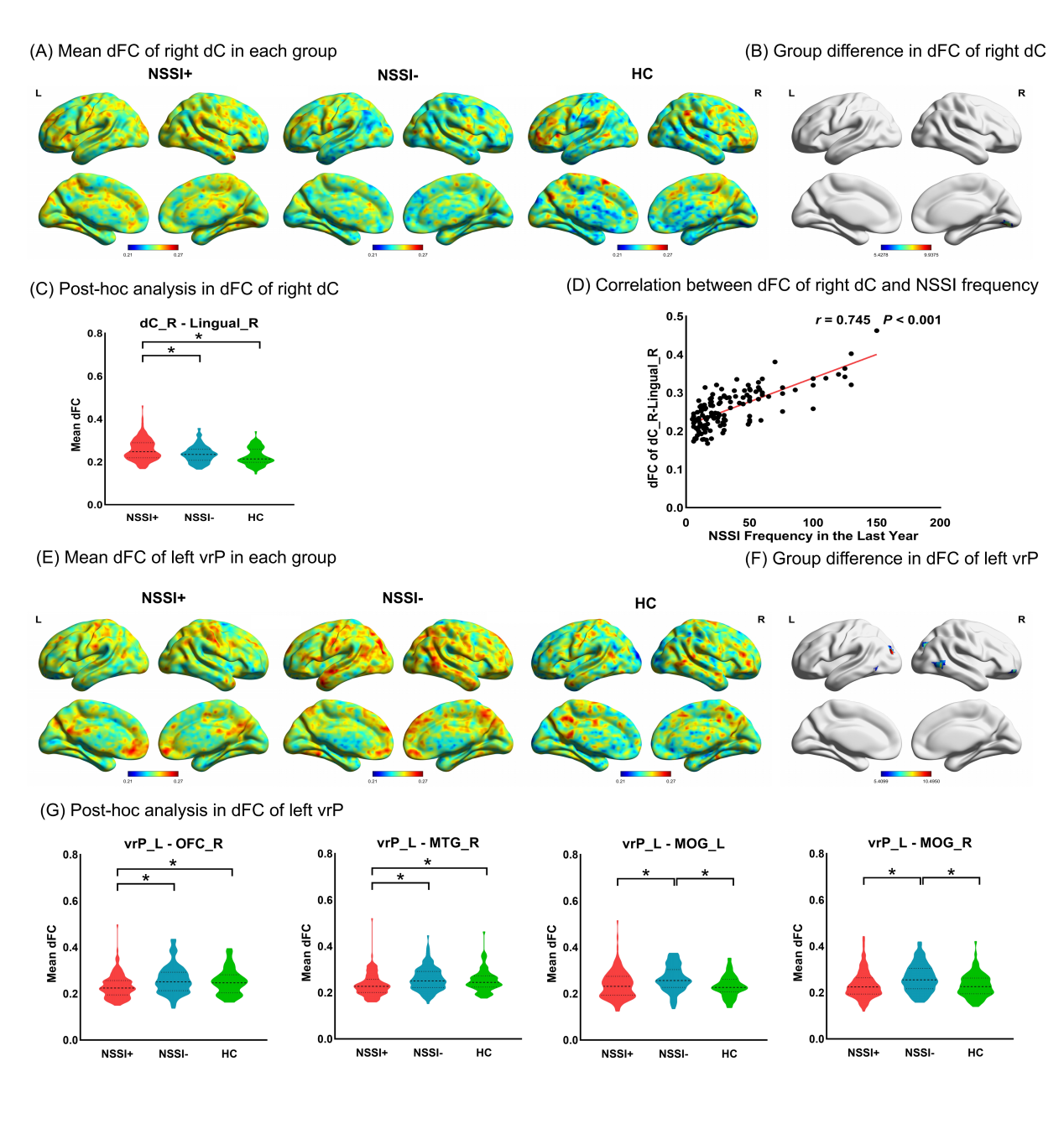


Figure S2. Profile Comparison and Association with NSSI in dFC in validation analysis (Window length: 60TRs). (A) The mean dFC map of right dC in each group. (B) Regions showing group difference in dFC of right dC. (C) Post-hoc analysis of regions with within-group differences in dFC of right dC. (D) Correlation between dFC of right dC and NSSI frequency. (E) The mean dFC map of left vrP in each group. (F) Regions showing group difference in dFC of left vrP. (G) Post-hoc analysis of regions with within-group differences in dFC of left vrP. *Bonferroni corrected, *P* < 0.05. dFC, dynamic functional connectivity; NSSI, non-suicidal self-injury; dC, dorsal caudate; vrP, ventral rostral putamen; OFC, orbitofrontal cortex; MTG, middle temporal gyrus; MOG, middle occipital gyrus.
